# Sample size determination with a pilot study

**DOI:** 10.1371/journal.pone.0262804

**Published:** 2022-02-15

**Authors:** Ben O’Neill

**Affiliations:** Research School of Population Health, Australian National University, Canberra, Australia; Indiana University Bloomington, UNITED STATES

## Abstract

We analyse standard confidence intervals for the mean of a finite population, with a view to sample size determination. We first consider a standard method for sample size determination based on the assumption of knowledge of the population variance parameter. We then consider the more realistic case where the population variance is treated as an unknown, and we derive a new method of sample size determination that accounts for the uncertainty in this parameter. We develop a sample size calculation method based on a preliminary sample of data which allows us to increase the accuracy of our inference by some specified amount with a specified level of confidence. We compare this method to the standard method.

## 1. Introduction

Statistical studies require data, and one is often presented with situations in which the amount of data is (at least partially) under the control of the experimenter. This gives rise to a trade-off problem for the experimenter—more data allows more accurate inference of parameters of interest, but it comes at a cost. The experimenter requires some means of balancing these considerations and this requires an understanding of the likely accuracy of the survey based on a given amount of data. This, briefly, is the problem of sample-size determination.

Adequate determination of sample sizes is particularly important in view of the consequence of failure. The collection of data has a resource cost (money, time, etc.) and there may also be other harms to consider from experimentation to obtain the relevant data (e.g., in medical studies there may be patient harm or sub-optimal treatment as part of experimentation). A sample which is too small can mean that the inferences from the statistical study are not of any practical value, and this is obviously something to be avoided. However, a sample which is too large means that the inferences from the statistical study are more precise than they need to be for practical purposes, and this often entails an unnecessary loss of resources or creation of harms.

In this paper we will consider the problem of sample size determination for a simple problem involving inference about the unknown mean of a finite population of values. We consider the case where there has been a pilot survey from the population, or some past survey from the population which can act as a pilot. Inference about the mean will be obtained using a classical confidence interval and accuracy is measured by the length of the interval. The goal will be to determine the minimum number of additional sample values which are needed to obtain some level of confidence about the length of the confidence interval which is the object of our inference.

Since the length of the confidence interval is affected by the variance of the population values this requires some consideration. [1] gives some practical advice on sample size determination, and notes that, in practice, the unknown variance parameter is estimated in these cases either from historical data, from the beliefs of experts, or from a pilot study (see pp. 189–190 for discussion). In the case of historical data or a pilot study there will be a set of actual data from the population. In the case of expert beliefs, it is possible to consider the information as a pseudo data-set, with specified sample size and moments representing the strength and nature of the expert beliefs. Each of these cases can therefore be considered in essentially the same way, by considering an existing data set from the population and using this to determine how many additional sample values are needed.

## 2. Sampling from a finite population

Confidence intervals for inference pertaining to the mean of a population are an established staple of introductory classical statistics. These inferential tools are frequently presented in introductory statistics textbooks in order to give students an appreciation of the nature of classical inference along with the law of large numbers and the central limit theorem (e.g., [2–4]). Derivation of confidence intervals for the mean and variance of a finite population can be found in [5] along with some useful moment results pertaining to this problem. One important part of this subject is to give an appreciation for the information contained in data from random sampling and the effect of sample size on accuracy. This can be considered through an examination of the required sample size for inferences at a certain level of accuracy, as determined by the length of appropriate confidence intervals.

Sampling problems are usually undertaken using the technique of simple random sampling without replacement. Mathematically, this method is most usefully expressed as the selection of values from an exchangeable population of values, usually considered to be embedded within an exchangeable superpopulation of random variables. Even for problems involving sampling from a finite population it is useful to regard the finite population as embedded in an infinite sequence of this kind (see [6]). This method allows us to consider any finite population as embedded in a wider sequence with simple properties, and it allows us to give an operational approach to the interpretation of model parameters (see e.g., [7], pp. 234–240).

In line with this approach, we consider an exchangeable superpopulation ***X*** = (*X*_1_, *X*_2_, *X*_3_,…) composed of real random variables. Since the sequence is exchangeable, this ensures that the elements of this sequence are independent and identically distributed (IID) conditional on the underlying empirical distribution of the sequence (see [8] for discussion). Since each element in the sequence is identically distributed we set $\mu =E(X_{i})$ and $\sigma^{2}=V(X_{i})$ as the common mean and variance, which are functions of the superpopulation values. (We will also use the kurtosis parameter *κ* which is defined by $\kappa \sigma^{4}=E({\left(X_{i}-\mu \right)}^{4})$.) The parameters can be expressed operationally as limits of functions from the sequence, having recourse to the laws of large numbers to justify this operational definition.

Within this superpopulation we will consider a finite population ***X***_*N*_ = (*X*_1_, *X*_2_,…,*X*_*N*_) and a smaller sample ***X***_*n*_ = (*X*_1_, *X*_2_,…,*X*_*n*_) taken from this population. (Since the sample is taken as the first n values of an exchangeable superpopulation it is implicitly a simple random sample without replacement from the finite population.) This sample contains *n* observed data values and the population vector contains an additional *N*−*n* unobserved values (the total population size is *N*). The sample mean and variance and the population mean and variance are defined respectively by:

$$\begin{array}{cc} {\bar{X}}_{n}=\frac{1}{n}\sum_{i=1}^{n}X_{i} & S_{n}^{2}=\frac{1}{n-1}\sum_{i=1}^{n}{\left(X_{i}-{\bar{X}}_{n}\right)}^{2},\\ {\bar{X}}_{N}=\frac{1}{N}\sum_{i=1}^{N}X_{i} & S_{N}^{2}=\frac{1}{N-1}\sum_{i=1}^{N}{\left(X_{i}-{\bar{X}}_{N}\right)}^{2}. \end{array}$$

(The reader should note that we incorporate Bessel’s correction into the population variance, which is contrary to the approach taken in some texts. This differs from some other treatments of sampling which use *N* as the denominator in the population variance. The reason we incorporate Bessel’s correction is that it makes sense to consider the finite population variance as an estimator of the superpopulation variance in this context. This correction ensures that the sample variance and population variance both have the same expected value *σ*^2^ and therefore function as unbiased estimators of this quantity.)

The sample moments ${\bar{X}}_{n}$ and $S_{n}^{2}$ can be used to make inferences about the mean and variance of the superpopulation or population using standard results in statistical theory. We will consider the case where we wish to use our sample data to make an inference about the unknown mean of the finite population. In particular, we will form a confidence interval for the mean ${\bar{X}}_{N}$ of the finite population. This interval is derived in [5] based on the central limit theorem. The 1−*α* level confidence interval for the population mean is given by:

$${\mathrm{CI}}_{N}(1-\alpha )=\left[{\bar{x}}_{n}\pm \frac{t_{\alpha /2,{DF}_{n}}}{\sqrt{n}}∙\sqrt{\frac{N-n}{N}}∙s_{n}\right]\;{DF}_{n}=\frac{2n}{\kappa -\left(n-3\right)/\left(n-1\right)}.$$

The degrees-of-freedom function *DF*_*n*_ depends on the kurtosis parameter *κ* (note that this is the raw kurtosis, not the excess kurtosis) which is unknown in practice. This unknown parameter can be replaced with an appropriate estimator to obtain the appropriate degrees-of-freedom and thereby obtain the confidence interval (see [5], Appendix II). This is the standard confidence interval form for inference about the finite population mean, except that textbooks on this topic operate under the assumption of a mesokurtic distribution where *κ* = 3 and so *DF*_*n*_ = *n*−1 (often without disclosing that assumption).

### Remark

Since the kurtosis parameter is unknown in practice, one might therefore wonder: why complain about the assumption that the population variance is known, when removal of this assumption then treats the population kurtosis as known? The value of the latter approach is that the kurtosis parameter only enters into the confidence interval through the degrees-of-freedom function so that it has a minor effect on the interval. We therefore gain something by treating the population variance as unknown, even though this gives us an interval framed in terms of the unknown kurtosis parameter.

The above confidence interval is also similar to the familiar confidence interval for inference about the parameter *μ*, except that we have an additional correction term which scales the length of the interval down to take account of the fact that we have knowledge of a non-zero proportion of the population values. As *N*→∞ the population becomes a superpopulation and the proportion of sampled values approaches zero, so that the correction term approaches unity. In this case we obtain the more familiar confidence interval for *μ* given by:

$${\mathrm{CI}}_{\infty }(1-\alpha )=\left[{\bar{x}}_{n}\pm \frac{t_{\alpha /2,{DF}_{n}}}{\sqrt{n}}∙s_{n}\right]\;{DF}_{n}=\frac{2n}{\kappa -\left(n-3\right)/\left(n-1\right)}.$$

We can regard the standard confidence interval for *μ* as a special case of the more general interval that allows for a finite population. We will therefore proceed on the basis of the more general case, with inference about *μ* being a special case.

Accuracy of our inference is determined by the length of the confidence interval, which is proportional to the sample standard deviation. The length of this interval is:

$$L_{n}=2∙\frac{t_{\alpha /2,{DF}_{n}}}{\sqrt{n}}∙\sqrt{\frac{N-n}{N}}∙S_{n}.$$

A shorter confidence interval corresponds to a more accurate inference and a wider interval corresponds to a less accurate inference. Since the sample standard deviation is unknown the length of the interval is unknown prior to seeing the data. Given that this length gives us a measure of accuracy, our goal will be to determine the minimum sample size that is sufficient to give some reasonable confidence that this length will be within an acceptable upper bound.

## 3. Sample size determination with known-variance

One unfortunate aspect of many theoretical treatments of sample size determination is the tendency to focus on the unrealistic case of a population with unknown mean but known variance. This leads to a standard form of confidence interval with accompanying theory which fails to take account of the uncertainty in the variance of the population. Methods for determining the required sample size for a particular level of accuracy (i.e., a confidence interval of a certain length) are treated in this way in standard texts on sampling theory such as [9] and [10]. These texts derive the required sample size based on a known population variance, which has the effect of understating the required size once we account for uncertainty in the variance. This is the way in which the subject is presented to early undergraduates, and the consequence is to give a highly unrealistic treatment of the problem. We consider this method in the present section.

In determining the sample size required for a particular level of accuracy, we are faced with the difficulty that the sample size is not fully determinative of the length of the interval. One simple method of dealing with this difficulty is to assume away the complication by treating the population variance as if it were a known quantity in the analysis. If the parameter *σ* is assumed to be known then the pivot statistic used to form the confidence interval is normally distributed instead of having a T-distribution. In this case we obtain the interval formulae:

$${\mathrm{CI}}_{N}^{\sigma }(1-\alpha )=\left[{\bar{x}}_{n}\pm \frac{z_{\alpha /2}}{\sqrt{n}}∙\sqrt{\frac{N-n}{N}}∙\sigma \right]\;{\mathrm{CI}}_{\infty }^{\sigma }(1-\alpha )=\left[{\bar{x}}_{n}\pm \frac{z_{\alpha /2}}{\sqrt{n}}∙\sigma \right].$$

This form of confidence interval is based on the unrealistic situation in which *μ* is unknown and *σ* is known. It is often presented in introductory statistical courses. Following [9] and [10] the standard sample size calculation for confidence intervals for a population mean assumes knowledge of the parameter *σ*. This uses the confidence interval above, based on the normal pivot statistic, which has length given by:

$$L_{n}^{\sigma }=2∙\frac{z_{\alpha /2}}{\sqrt{n}}∙\sqrt{\frac{N-n}{N}}∙\sigma .$$

Setting $L_{n}^{\sigma }=2H$ (giving half-length *H*) and solving for *n* gives us:

$$n=\frac{1}{1/n_{0}+1/N}\;n_{0}=\frac{z_{\alpha /2}^{2}∙\sigma^{2}}{H^{2}}.$$

This is the sample size required to achieve a confidence interval with length 2*H* for inference about the finite population mean. (This formula will generally yield a non-integer value. To find the required sample size we round up to the next integer.) As *N*→∞ we have *n*→*n*_0_ so that *n*_0_ represents the required sample size for inference about the superpopulation mean.

This method assumes that the variance parameter is known, but in practice this parameter is often taken from a pilot sample or historical data, and the sample variance from the pilot sample is substituted as the parameter *σ*. Hence, what is really occurring is that the analyst treats an estimate of the variance parameter as if it were perfectly accurate. To accommodate this case, we will suppose we have an existing sample of *n* objects and we want to sample more data points to reduce the length of our confidence interval, relative to the length of the confidence interval obtained from the preliminary sample. The sample variance in the original sample is taken to be a perfect estimator of the variance parameter, so that it is treated as known. To do this we will choose some number 0≤*k*≤*N*−*n* of additional data points. We will consider the ratio of the length values for the confidence intervals formed from the full data set and the preliminary data set. This length ratio is given by:

$$\frac{L_{n+k}^{\sigma }}{L_{n}^{\sigma }}=R(k)\equiv \sqrt{\frac{N-n-k}{N-n}∙\frac{n}{n+k}}\;\mathrm{for}\;\mathrm{all}\;0\leq k\leq N-n.$$

This is a decreasing function of *k* which means that as we obtain more data the size of the confidence interval decreases. This follows from the fact that we are using a fixed variance value. By specifying the ratio 0<*R*<1 we can easily determine the required number *k* of additional data points that are needed to reduce the present length of the confidence interval from the preliminary data.

### Example 1

We consider an example used in [10] (pp. 16, 37) using data taken from and aerial survey of a caribou population. Suppose we have a preliminary sample of *n* = 15 data points from a population of *N* = 286. The full sample is given by:

$$x_{n}=(1,50,21,98,2,36,4,29,7,15,86,10,21,5,4).$$

From this sample we obtain the statistics ${\bar{x}}_{n}=25.933$ and *s*_*n*_ = 30.316. If we assume that *σ* = *s*_*n*_ = 30.316 then the 90% confidence interval for the finite population mean ${\bar{X}}_{N}$ from our preliminary sample is:

$$\mathrm{CI}(0.90)=25.933\pm \frac{1.645}{\sqrt{15}}∙\sqrt{\frac{271}{286}}∙30.316=25.933\pm 12.533=[13.400,38.466].$$

We want to know the number of data points required to estimate the population mean with sufficient accuracy to be within ±2000 of the true population total. This means that the required interval half-length for the population mean is *H* = 2000/*N* = 2000/286 = 6.993 so that the required length ratio is:

$$R=\frac{H}{12.533}=\frac{6.993}{12.533}=0.5580.$$

**Fig 1:**
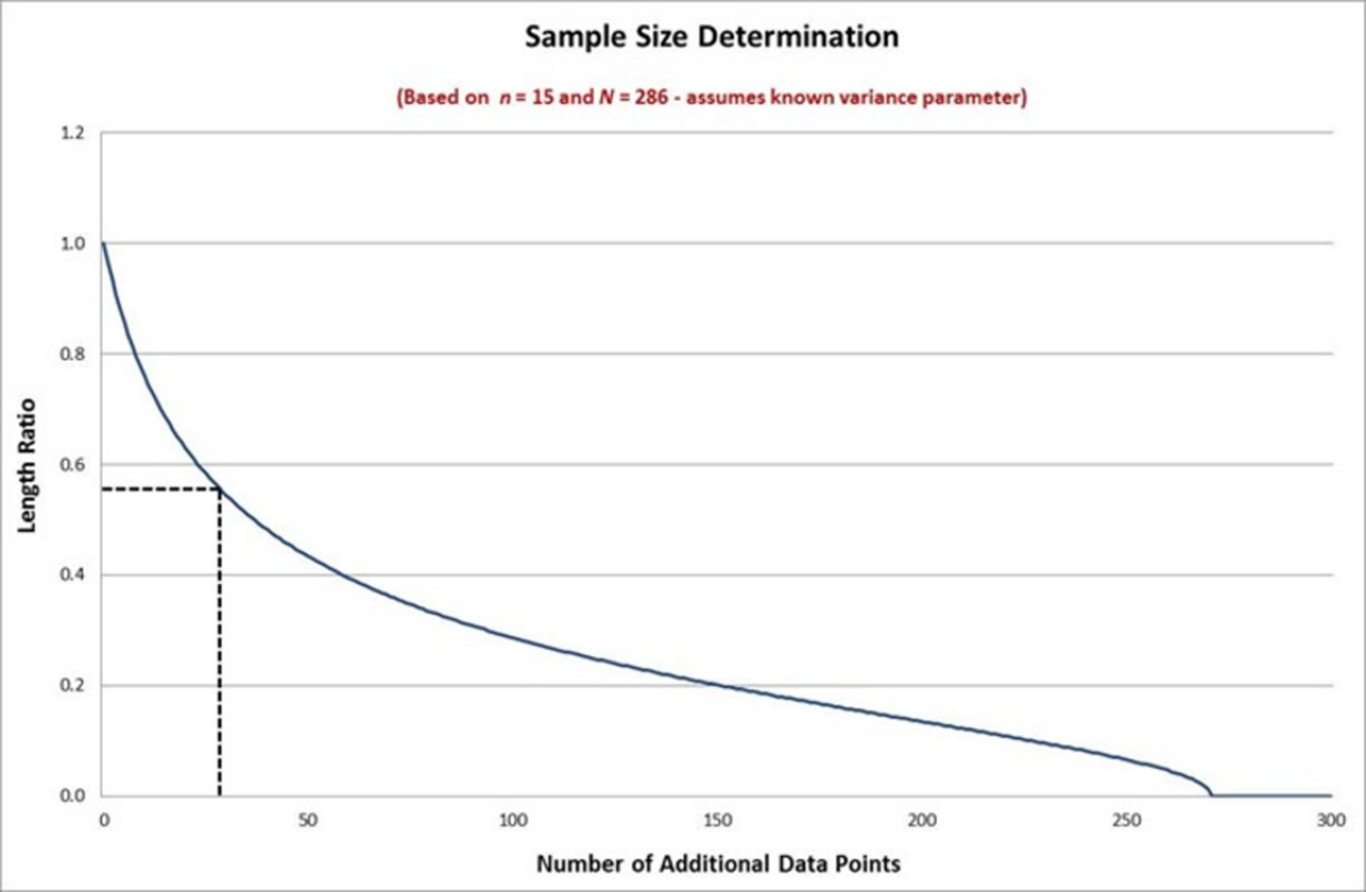
Sample size determination using the standard method (variance known).

The first integer value satisfying this upper bound is *k* = 29 where we have *R*(*k*) = 0.5517. This means that we need an additional *k* = 29 data points for our total sample bringing us to *n*+*k* = 44 data points out of *N* = 286. Fig 1 above shows how the ratio of the lengths decreases as *k* increases. When *k* = 0 the length ratio is unity, and when *k* = 271 all the remaining objects in the population have been sampled so that the length ratio is zero. Between these extremes the length of the interval reduces commensurate with the information given by the additional data. The sample size derived in the present case can be seen on the figure.

## 4. An improved sample size determination method

We will again consider the case where we have a preliminary sample of *n* data points from a population of *N* data points. We will again choose some number 0≤*k*≤*N*−*n* of additional data points in an attempt to obtain an inference with some required level of accuracy, which we determine through the length of our confidence interval. However, we will now take account of the uncertainty the variance by dealing with the confidence intervals using the sample standard deviation.

To determine the appropriate value of *k* for our inference we will again consider the lengths of the intervals. The ratio of the lengths of the total sample of *n*+*k* values to the existing sample of *n* values is now given by:

$$\frac{L_{n+k}}{L_{n}}=R(k)∙\frac{t_{\alpha /2,{DF}_{n+k}}}{t_{\alpha /2,{DF}_{n}}}∙\frac{S_{n+k}}{S_{n}}.$$

Unlike in the standard method, this length ratio is now a random variable, and this means that we will want to determine its distribution. To determine the distribution of the length ratio we consider the distribution of the ratio of nested sample variances in the above ratio form. We use the following asymptotic distributional result (see Appendix):

$$\frac{S_{n+k}^{2}}{S_{n}^{2}}\overset{Approx}{∼}\frac{n+kF({DF}_{C},{DF}_{n})-1}{n+k-1}\;{DF}_{C}=\frac{2k(n+k)}{2(n+k)+(\kappa -3)(n+k-2+1/n)}.$$

(The notation *F*(*DF*_*C*_, *DF*_*n*_) here refers to Snedecor’s F distribution with numerator degrees-of-freedom *DF*_*C*_ and denominator degrees-of-freedom *DF*_*n*_.) This distributional is exact for normal values, or holds as an asymptotic distribution by appeal to the central limit theorem as *n*→∞ and *k*→∞. It holds as an adequate approximation when *n* and *k* are large. This gives us an implied distributional form for the length ratio bound, which we can use to determine the required sample size for a given level of accuracy.

In the standard method which assumes a known variance parameter our ratio bound was fully determined by the relevant population and sample sizes so that we could specify the required length ratio directly. Since our length ratio is now a random variable we must refine what it means to specify the required accuracy. In addition to specifying a required length ratio we will now also specify some confidence level 1−*δ* such that the required length ratio bound holds at the specified confidence level. (Do not confuse the two confidence levels: the confidence interval of interest has confidence level 1−*α* and the sample size determination uses confidence level 1−*δ*.)

Using the relevant critical point of the F distribution, we have the following approximation result which holds when *n* and *k* are large (or when the data are close to normal):

$$1-\delta \approx P\left(\frac{S_{n+k}^{2}}{S_{n}^{2}}\leq \frac{n+kF_{\delta ,{DF}_{C},{DF}_{n}}-1}{n+k-1}\right)$$

$$=P\left({\left(\frac{L_{n+k}}{L_{n}}\right)}^{2}\leq {R(k)}^{2}∙\frac{t_{\alpha /2,{DF}_{n+k}}^{2}}{t_{\alpha /2,{DF}_{n}}^{2}}∙\frac{n+kF_{\delta ,{DF}_{C},{DF}_{n}}-1}{n+k-1}\right)$$

$$=P\left(\frac{L_{n+k}}{L_{n}}\leq R(k)∙\frac{t_{\alpha /2,{DF}_{n+k}}}{t_{\alpha /2,{DF}_{n}}}∙\sqrt{\frac{n+kF_{\delta ,{DF}_{C},{DF}_{n}}-1}{n+k-1}}\right)$$

$$=P\left(\frac{L_{n+k}}{L_{n}}\leq R_{\delta }(k)\right),$$

where we define the length-ratio bound function *R*_*δ*_ by:

$$R_{\delta }(k)=R(k)A_{\delta }(k)\;A_{\delta }(k)\equiv \frac{t_{\alpha /2,{DF}_{n+k}}}{t_{\alpha /2,{DF}_{n}}}∙\sqrt{\frac{n+kF_{\delta ,{DF}_{C},{DF}_{n}}-1}{n+k-1}}.$$

Hence, with confidence level 1−*δ* we know that using *k* additional data points will reduce the length of the confidence interval by the multiplicative factor *R*_*δ*_(*k*) or less. It is then easy to choose *k* to satisfy some required reduction in length, at some specified confidence level.

The function *R*_*δ*_ is also implicitly dependent on the kurtosis parameter *κ* through the degrees-of-freedom quantities *DF*_*C*_, *DF*_*n*_ and *DF*_*n*+*k*_. In practice we will either estimate this from the data in the pilot survey or assume the value of this parameter to be known. (In the special case where we are willing to assume a mesokurtic distribution we have *κ* = 3 so that the degrees-of-freedom quantities become *DF*_*C*_ = *k*, *DF*_*n*_ = *n*−1 and *DF*_*n*+*k*_ = *n*+*k*−1.)

### Example 2

**Fig 2:**
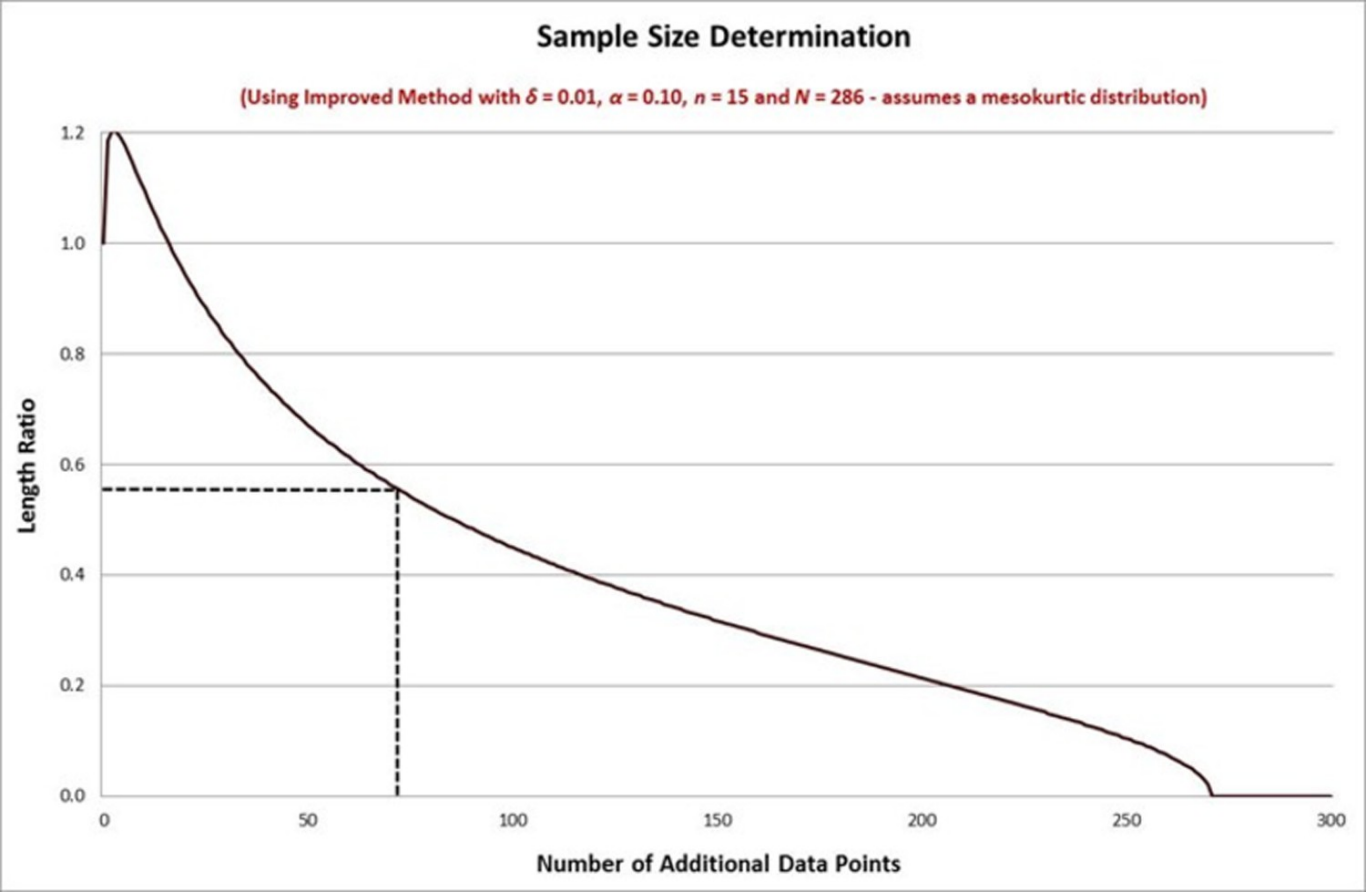
Sample size determination using the improved method (variance unknown).

Continuing Example 1, we will now apply our new sample size determination method to the previous example. Recall that we want to reduce the length of the confidence interval from the preliminary data set by the factor *R* = 0.5580 by taking additional data points. If we want this reduction to occur with 99% confidence then we set *δ* = 0.01 and examine the length-ratio boundary function at this confidence level. (In this case we will simplify our problem by assuming a mesokurtic distribution, so that *κ* = 3.) The first integer value satisfying the required upper bound is *k* = 72 where we have *R*_*δ*_(*k*) = 0.5554 (shown in the plot below). This means that we need an additional *k* = 72 data points for our total sample bringing us to *n*+*k* = 87 data points out of *N* = 286. This is substantially higher than what we estimated in Example 1 when we used the standard method which assumed a known variance parameter. The reason for this is that we are now taking into account the fact that the sample variance will change as we obtain new data and this additional uncertainty with affect the interval length. Fig 2 below shows the length ratio as a function of the number of additional data points, taking into account the uncertainty in the population variance. The additional uncertainty means that we now require more data points than in Example 1.

The full plot shows how the ratio of the lengths changes as *k* increases. Unlike in the case of the standard method this is no longer a decreasing function in this case (it is quasi-concave). When *k* = 0 the length ratio is unity, but as *k* increases the bound initially increases before starting to decrease again. The reason for this is that a small number of additional data points increases the uncertainty in the sample variance ratio, and can plausibly lead to an increase in the length of the confidence interval of interest, since the additional data points may actually increase the sample standard deviation more than they reduce the weight applied to this sample standard deviation in the interval. (Since we use a 90% confidence interval, we look at the extreme that occurs when the quantile of the variance ratio in the top 10% of its distribution.) As more additional data points are taken, this effect is overcome by the reduction in length due to a larger sample size and the function starts to decrease. When *k* = 271 all the objects in the population have been sampled in the full sample so that the length ratio is zero.

(In some cases we may not want to use the preliminary data in our new confidence interval.) In this case the length ratio of the preliminary interval and the final interval is given by:

$$\frac{L_{n:k}}{L_{n}}=\frac{t_{\alpha /2,{DF}_{k}}}{t_{\alpha /2,{DF}_{n}}}∙\sqrt{\frac{N-k}{N-n}∙\frac{n}{k}}∙\frac{S_{n:k}}{S_{n}}\;\mathrm{for}\;\mathrm{all}\;0\leq k\leq N.$$

(The quantity $S_{n:k}^{2}$ in this equation is the standard deviation of the additional sample of *k* data points, without inclusion of the preliminary sample of *n* data points.) We use the following asymptotic distributional result (see Appendix):

$$\frac{S_{n:k}^{2}}{S_{n}^{2}}\overset{Approx}{∼}F({DF}_{k},{DF}_{n})\;{DF}_{k}=\frac{2k}{\kappa -\left(k-3\right)/\left(k-1\right)}.$$

This leads to the length-ratio boundary function:

$$R_{\delta }^{*}(k)\equiv \frac{t_{\alpha /2,{DF}_{k}}}{t_{\alpha /2,{DF}_{n}}}\sqrt{\frac{N-k}{N-n}∙\frac{n}{k}∙F_{\delta ,{DF}_{k},{DF}_{n}}}\;\mathrm{for}\;\mathrm{all}\;0\leq k\leq N.$$

This would be used as an alternative when we do not want to include the preliminary data in our new confidence interval and we are selecting our new data using simple random sampling from the entire population.)

## 5. Comparison of methods

The present method improves on the standard method that assumes the variance parameter to be known. The improvement follows directly by incorporating the uncertainty in the variance estimate. Except for very low confidence levels this leads to larger required sample sizes than are determined under the standard method, with the higher sample size required in order to account for the additional uncertainty in the absence of this assumption.

It is worth noting that both of these methods rely on distributional approximations based on the central limit theorem, so that both methods are susceptible to problems in dealing with small samples or heavy-tailed distributions. This can be regarded as an inherent limitation on both methods, which is unlikely to be alleviated unless there is information on the form of distribution of the superpopulation. Nevertheless, the present method is no worse in this regard than the standard sample size determination method, and can be regarded as an improvement insofar as it removes one unrealistic assumption in that analysis.

**Fig 3:**
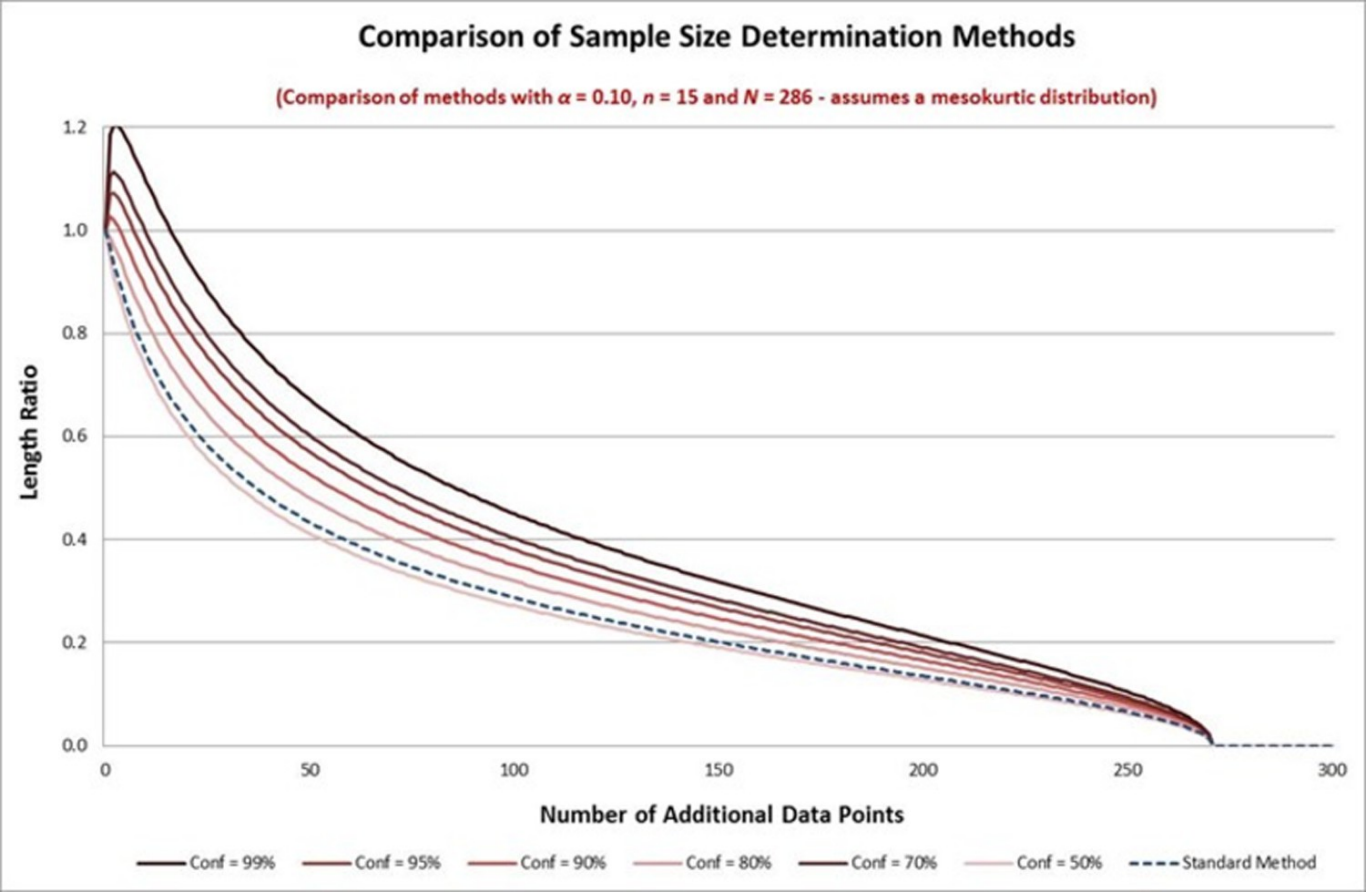
Comparison of sample size determination methods.

It is easy to compare the length ratio functions of the two methods by looking at the function *A*_*δ*_. This function determines the difference in the length ratio bound under the two methods. It is easy to show that this is a decreasing function of *δ* so that higher levels of confidence (lower values of *δ*) for the required sample size lead to a higher length ratio bound under the method set out in this paper. To see the difference in the length bounds for various different confidence levels, and in comparison to the standard method, we show the length ratio bound using the values of *n* and *N* in Example 1 in Fig 3 below. We can see that the length ratio bound function reduces as we reduce our confidence level. We note that our figure here shows values of *k* = *N*−*n* that begin low, notwithstanding that we use asymptotic distributions.

**Fig 4:**
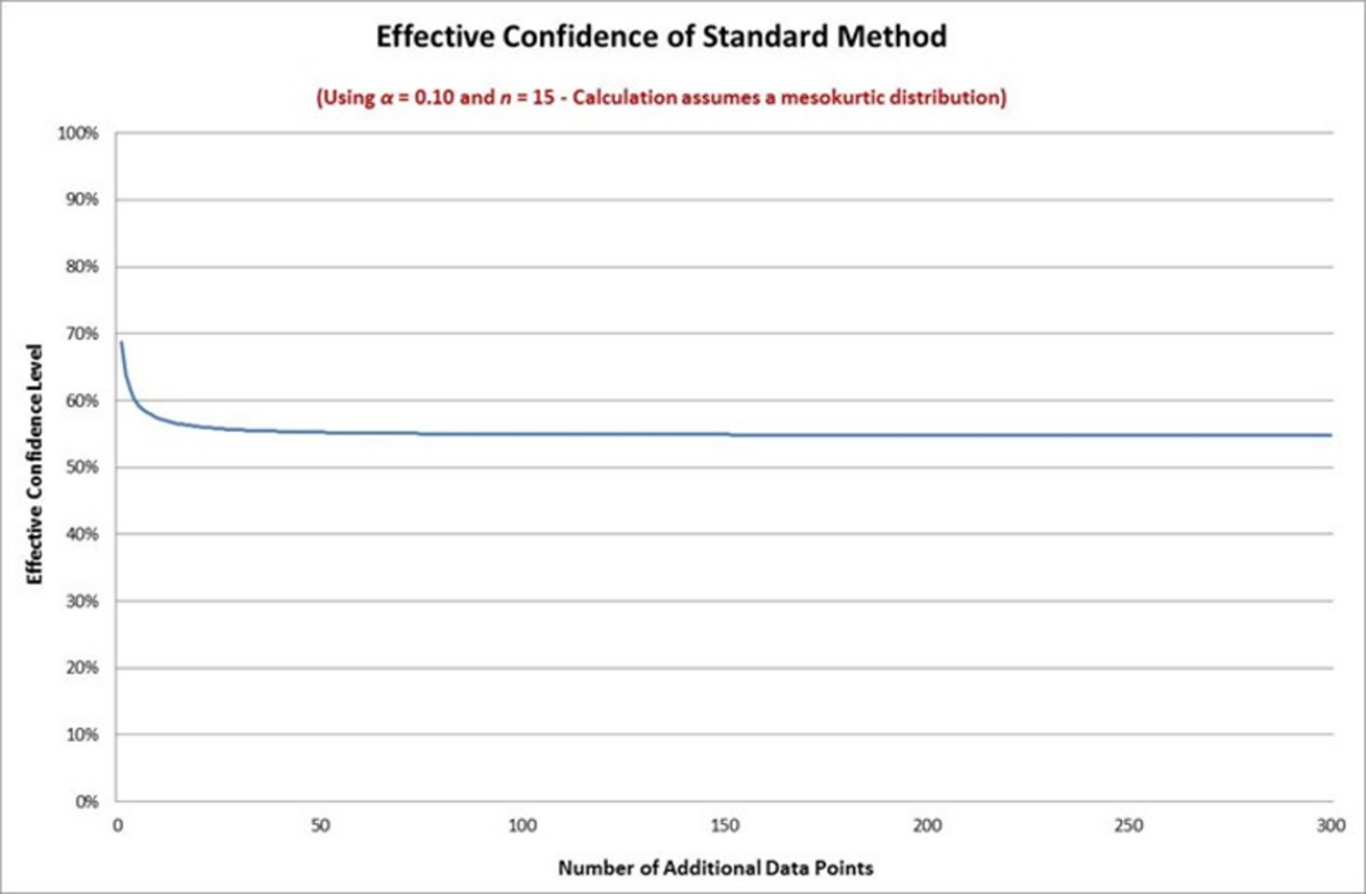
Effective confidence of standard method.

In Fig 3 we can see that the standard method gives us an effective confidence that is somewhere between the 50% and 70% levels. We can be more precise that this by plotting the effective confidence level of the method, defined by setting *A*_*δ*_(*k*) = 1 and solving for *δ*. (This effective confidence level does not depend on *N*.) (This also requires specification of the kurtosis parameter, which can either be estimated from the pilot sample or specified by assumption.) The effective confidence level for a mesokurtic distribution is shown in Fig 4 below for all values of *k*≥1 using the number of preliminary data points in Example 1.

Fig 4 shows that the effective confidence level is about 55% with exact level depending on *k*. This means that the standard method would not give a high level of confidence of actually achieving the desired reduction in the length of the confidence interval—in fact, this would occur slightly more often than half the time. The remainder of the time the new data points in the sample would increase the sample standard deviation so that the new confidence interval is wider than would have been expected under the standard method. This illustrates a major drawback in the standard method—by taking the variance parameter to be known it effectively ignores the sample variance estimator, and thereby ignores the possibility that the sample variance may increase once the new data is taken.

We can see that the effective confidence of the standard method approaches a limiting value as *k*→∞. Since $\underset{k\to \infty }{\lim}F_{\delta ,{DF}_{C},{DF}_{n}}={DF}_{n}/\chi_{1-\delta ,{DF}_{n}}^{2}$ and $\underset{k\to \infty }{\lim}t_{\alpha /2,{DF}_{n+k}}=z_{\alpha /2}$ we have:

$$A_{\delta }(\infty )=\underset{k\to \infty }{\lim}A_{\delta }(k)=\frac{z_{\alpha /2}}{t_{\alpha /2,{DF}_{n}}}∙\sqrt{\frac{{DF}_{n}}{\chi_{1-\delta ,{DF}_{n}}^{2}}}.$$

Hence, taking *A*_*δ*_(∞) = 1 we have the equation:

$$\frac{\chi_{1-\delta ,{DF}_{n}}^{2}}{{DF}_{n}}=\frac{z_{\alpha /2}^{2}}{t_{\alpha /2,{DF}_{n}}^{2}}.$$

Solving for *δ* we obtain the limiting value of the effective confidence level. Using the values from Example 1 (assuming a mesokurtic distribution) we have a limiting confidence level of 54.78%. This is the limiting value for the plot in Fig 4.

Since the standard method is based on the assumption that *σ* is known, it is also instructive to see what happens when the value of *n* is large, so that we have a very good inference about the parameter *σ* (i.e., it is almost known). Setting the value *τ*≡2/(*κ*−1) it can be shown that $\underset{n\to \infty }{\lim}F_{\delta ,{DF}_{C},{DF}_{n}}=\chi_{\delta ,\tau k}^{2}/\tau k$ so that:

$$\underset{n\to \infty }{\lim}A_{\delta }(k)=\underset{n\to \infty }{\lim}\sqrt{\frac{n+\chi_{\delta ,\tau k}^{2}/\tau -1}{n+k-1}}=1.$$

This means that as *n*→∞ the length ratio bound calculated under the present method (at any confidence level) converges towards the length ratio for the standard method. This accords with our intuitive understanding of the estimation process, since *n*→∞ means that we are gaining perfect knowledge of the parameter *σ*, which is the assumption underlying the standard sample size determination method.

As a caveat to the above analysis, we note that our figures consider values of *k* = *N*−*n* that begin at zero and go upward spanning low values. Our figures also use *n* = 15, which is not a particularly high value. Since we use asymptotic distributions derived from the central limit theorem for the variance ratios in our analysis, the particular results shown in the figures are likely to be accurate only when the underlying distribution of the data is close to a normal distribution. Nevertheless, even with this shortcoming (which really cannot be avoided), our analysis is still likely to be an improvement over the standard sample-size analysis derived from the Z test, since it now accounts for uncertainty in the sample variance.

## 6. Conclusion

The present paper improves on the standard method for sample size determination using a preliminary sample in a standard single sample confidence interval problem. This improved method uses the distribution of the nested sample variance ratio to establish the length ratio bound that can be obtained with a given level of confidence. This allows the user to determine the number of additional data points required to obtain a given length bound for the confidence interval with a pre-specified level of confidence.

It would be possible to extend the present technique to obtain length ratio bound functions in other standard confidence interval problems involving mean comparison for multiple populations or stratified sampling from a single population. This could be done using the Welch-Satterwaite approximation that is used in the construction of these intervals. This would give a greater level of confidence of adequate sample size than techniques which rely on an assumption of known variance.

## Appendix: Approximating distributions

Here we show some approximate distributional results adapted from [5] that are used in the main body of the paper. These results are based on use of the central limit theorem, so they hold exactly when the data is normal, or approximately when *n* and *k* are large.

### Result 1

For large *n* and *k* we have:

$$\frac{S_{n:k}^{2}}{S_{n}^{2}}\overset{Approx}{∼}F({DF}_{k},{DF}_{n})\;\frac{S_{n+k}^{2}}{S_{n}^{2}}\overset{Approx}{∼}\frac{n+kF({DF}_{C},{DF}_{n})-1}{n+k-1}.$$

where:

$${DF}_{k}=\frac{2k}{\kappa -\left(k-3\right)/\left(k-1\right)}\;{DF}_{C}=\frac{2k(n+k)}{2(n+k)+(\kappa -3)(n+k-2+1/n)}.$$

Proof of Result 1

The first distributional approximation uses Result 14 of [5] (p. 285), which shows that:

$$\frac{S_{n}^{2}}{\sigma^{2}}\overset{Approx}{∼}\frac{ChiSq({DF}_{n})}{{DF}_{n}}\;\frac{S_{n:N}^{2}}{\sigma^{2}}\overset{Approx}{∼}\frac{ChiSq({DF}_{n:N})}{{DF}_{n:N}}.$$

where:

$${DF}_{n}=\frac{2n}{\kappa -\left(n-3\right)/\left(n-1\right)}\;{DF}_{n:N}=\frac{2(N-n)}{\kappa +\left(N-n-3\right)/\left(N-n-1\right)}.$$

Taking *N* = *n*+*k* the formula for *DF*_*n*:*N*_ reduces to the one in the theorem, and we have:

$$\frac{S_{n:k}^{2}}{S_{n}^{2}}\overset{Approx}{∼}\frac{ChiSq({DF}_{k})}{{DF}_{k}}/\frac{ChiSq({DF}_{n})}{{DF}_{n}}∼F({DF}_{k},{DF}_{n}).$$

The second distributional approximation is based on Result 15 of [5] (p. 286), which shows that:

$$\frac{S_{N}^{2}}{S_{n}^{2}}\overset{Approx}{∼}\frac{n-1}{N-1}+\frac{N-n}{N-1}∙\frac{1}{F({DF}_{n},{DF}_{C})},$$

where:

$${DF}_{n}=\frac{2n}{\kappa -\left(n-3\right)/\left(n-1\right)}\;{DF}_{C}=\frac{2(N-n)}{2+(\kappa -3)(1-2/N+1/Nn)}.$$

We note that the inverse of an F-distributed random variable is also an F-distributed random variable with its degrees-of-freedom parameters reversed, so *F*(*DF*_*C*_, *DF*_*n*_)~1/*F*(*DF*_*C*_, *DF*_*n*_). Taking *N* = *n*+*k* the formula for *DF*_*C*_ reduces to the one in the theorem, and we have:

$$\frac{S_{N}^{2}}{S_{n}^{2}}\overset{Approx}{∼}\frac{n-1}{n+k-1}+\frac{k}{n+k-1}∙\frac{1}{F({DF}_{n},{DF}_{C})}$$

$$∼\frac{n-1}{n+k-1}+\frac{k}{n+k-1}∙F({DF}_{C},{DF}_{n})$$

$$∼\frac{n+kF({DF}_{C},{DF}_{n})-1}{n+k-1}.$$

This establishes the distributional results that were to be shown.
